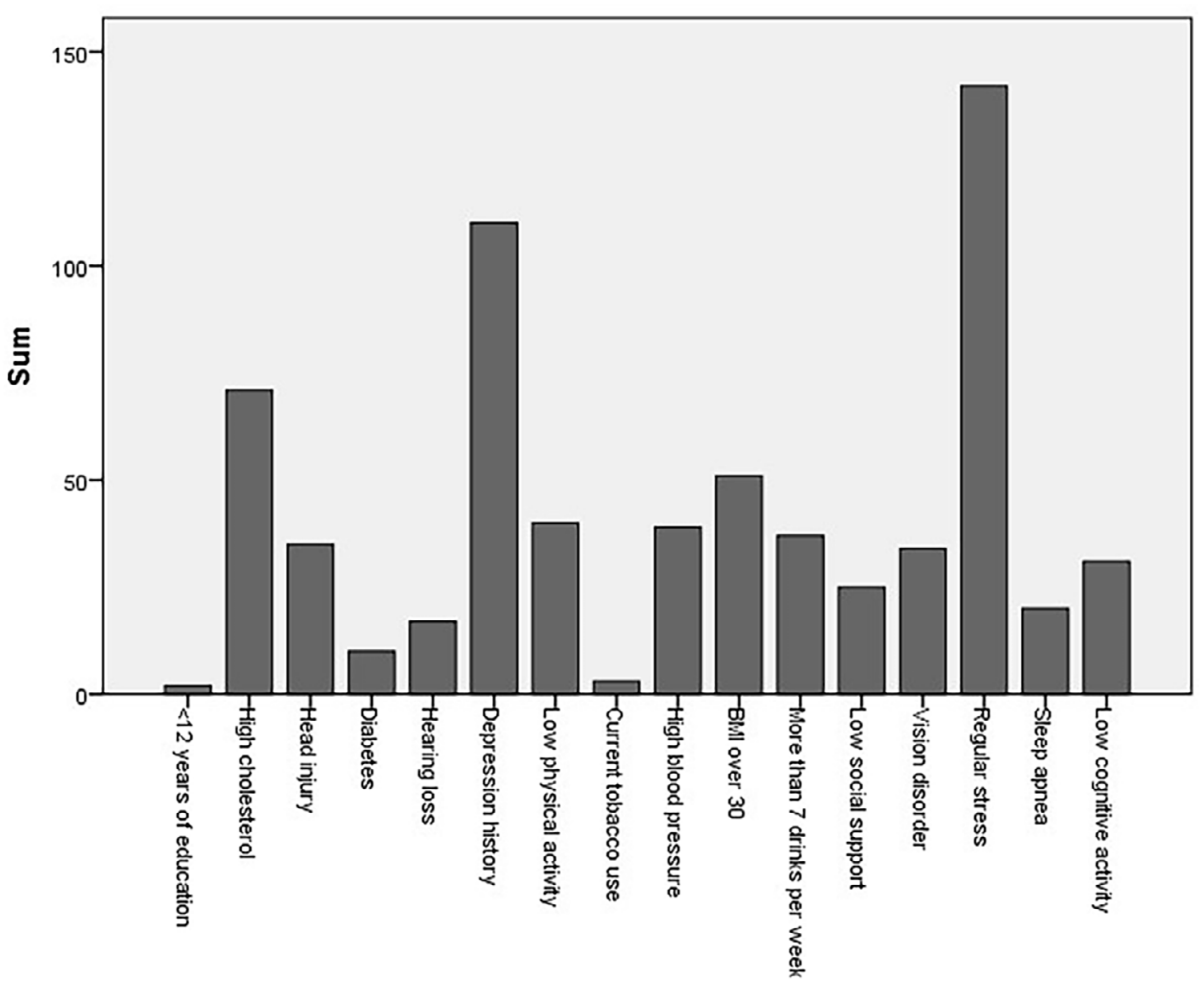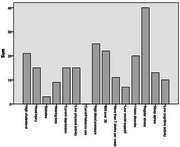# Impact of one year of lifestyle change on Alzheimer's risks and the role of dementia risk and menopause stage on longitudinal memory scores

**DOI:** 10.1002/alz70860_104614

**Published:** 2025-12-23

**Authors:** Jessica ZK Caldwell

**Affiliations:** ^1^ Cleveland Clinic Lou Ruvo Center for Brain Health, Las Vegas, NV, USA

## Abstract

**Background:**

Modifiable factors account for 45% of current dementia cases, and midlife is a critical time to reduce risks. Little research examines modifiable risks in women, or how risks relate to midlife memory and menopause. This project assessed women's menopause stage, dementia risk, and memory at baseline (BL) and 1‐year after (Y1) receiving risk reduction recommendations.

**Method:**

We analyzed data from women with a family history of dementia at BL (*N* = 280; age M=53.1; education M=16.7; 88% White; 93% Non‐Hispanic) and Y1 (*N* = 96; age M=55.5; education = 16.6; 88% White; 95% Non‐Hispanic). Total risk score was the sum (0/1 absent/present) of: <12 years of education; self‐reported history of high cholesterol, diabetes, hypertension, head injury, hearing loss, vision disorder, and depression; current lack of social support, tobacco use, >7 alcoholic beverages per week, and low physical activity; and baseline BMI>30. At Y1, risk scores used baseline plus new diagnoses for history variables, and Center for Epidemiologic Studies of Depression Scale for depression. Menopause stage was assessed via self‐report (pre‐ or post‐menopause), and for a subset, Stages of Reproductive Aging Workshop (STRAW) interview. Supplementary analyses added self‐reported stress, low cognitive activity, and sleep apnea to risk. Wilcoxon signed ranks examined differences between BL and Y1 risk, and Mann‐Whitney tests pre/post menopause differences. Spearman correlation assessed relationship between risk and memory.

**Result:**

Common BL risks included depression, stress, high cholesterol, and BMI>30. Y1risks were similar, with the addition of high blood pressure. At Y1, women showed lower overall total and supplemented risk scores (Z=‐3.98, *p* < .001; Z=‐2.855, *p* = .004). Greater BL risk scores were marginally related to poorer Y1 verbal learning and memory (*r* = ‐0.27, *p* = .07; r=‐0.27, *p* = .082). Postmenopausal women showed worse BL memory (FNAME: Z=‐2.98, *p* = .003; RAVLT learning: Z=‐2.08, *p* = .038; RAVLT delay: ‐1.62, *p* = .11) and marginally worse Y1 memory (FNAME: Z=‐1.69, *p* = .09), though only worse BL learning (‐2.10, *p* = .035) was significantly linked to STRAW post‐menopause.

**Conclusion:**

Vascular and psychosocial risks were common in women, and personalized lifestyle recommendations reduced total risks over one year. Nonetheless, memory differences in postmenopausal women and those with greater baseline dementia risks at midlife suggest need for further research on effective intervention.